# Low free 25-hydroxyvitamin D and high vitamin D binding protein and parathyroid hormone in obese Caucasians. A complex association with bone?

**DOI:** 10.1371/journal.pone.0192596

**Published:** 2018-02-28

**Authors:** Elisa Saarnio, Minna Pekkinen, Suvi T. Itkonen, Virpi Kemi, Heini Karp, Kaisa K. Ivaska, Juha Risteli, Marja-Kaisa Koivula, Merja Kärkkäinen, Outi Mäkitie, Harri Sievänen, Christel Lamberg-Allardt

**Affiliations:** 1 Calcium Research Unit, Department of Food and Nutrition, University of Helsinki, Helsinki, Finland; 2 Folkhälsan Institute of Genetics, Folkhälsan Research Center, Helsinki, Finland; 3 Children’s Hospital, Helsinki University Central Hospital and University of Helsinki, Helsinki, Finland; 4 Department of Cell Biology and Anatomy, Institute of Biomedicine, University of Turku, Turku, Finland; 5 Department of Clinical Chemistry, University of Oulu, Oulu, Finland; 6 Northern Finland Laboratory Centre Nordlab, Oulu, Finland; 7 Medical Research Center, Oulu, Finland; 8 Tykslab, the Hospital District of Southwest Finland, Turku, Finland; 9 Department of Clinical Chemistry, University Hospital Turku, Turku, Finland; 10 The UKK Institute for Health Promotion Research, Tampere, Finland; University Sains Malaysia, MALAYSIA

## Abstract

**Background:**

Studies have shown altered vitamin D metabolism in obesity. We assessed differences between obese and normal-weight subjects in total, free, and bioavailable 25-hydroxyvitamin D (25(OH)D, 25(OH)D_Free_, and 25(OH)D_Bio_, respectively), vitamin D binding protein (DBP), parathyroid hormone (PTH) and bone traits.

**Methods:**

595 37-47-year-old healthy Finnish men and women stratified by BMI were examined in this cross-sectional study. Background characteristic and intakes of vitamin D and calcium were collected. The concentrations of 25(OH)D, PTH, DBP, albumin and bone turnover markers were determined from blood. 25(OH)D_Free_ and 25(OH)D_Bio_ were calculated. pQCT was performed at radius and tibia.

**Results:**

Mean±SE (ANCOVA) 25(OH)D_Free_ (10.8±0.6 vs 12.9±0.4 nmol/L; P = 0.008) and 25(OH)D_Bio_ (4.1±0.3 vs 5.1±0.1 nmol/L; P = 0.003) were lower in obese than in normal-weight women. In men, 25(OH)D (48.0±2.4 vs 56.4±2.0 nmol/L, P = 0.003), 25(OH)D_Free_ (10.3±0.7 vs 12.5±0.6 pmol/L; P = 0.044) and 25(OH)D_Bio_ (4.2±0.3 vs 5.1±0.2 nmol/L; P = 0.032) were lower in obese. Similarly in all subjects, 25(OH)D, 25(OH)D_Free_ and 25(OH)D_Bio_ were lower in obese (P<0.001). DBP (399±12 vs 356±7mg/L, P = 0.008) and PTH (62.2±3.0 vs 53.3±1.9 ng/L; P = 0.045) were higher in obese than in normal-weight women. In all subjects, PTH and DBP were higher in obese (P = 0.047and P = 0.004, respectively). In obese women, 25(OH)D was negatively associated with distal radius trabecular density (R^2^ = 0.089, P = 0.009) and tibial shaft cortical strength index (CSI) (R^2^ = 0.146, P = 0.004). 25(OH)D_Free_ was negatively associated with distal radius CSI (R^2^ = 0.070, P = 0.049), radial shaft cortical density (CorD) (R^2^ = 0.050, P = 0.045), and tibial shaft CSI (R^2^ = 0.113, P = 0.012). 25(OH)D_Bio_ was negatively associated with distal radius CSI (R^2^ = 0.072, P = 0.045), radial shaft CorD (R^2^ = 0.059, P = 0.032), and tibial shaft CSI (R^2^ = 0.093, P = 0.024).

**Conclusions:**

The associations between BMI and 25(OH)D, 25(OH)D_Free_, and 25(OH)D_Bio_, DBP, and PTH suggest that obese subjects may differ from normal-weight subjects in vitamin D metabolism. BMI associated positively with trabecular bone traits and CSI in our study, and slightly negatively with cortical bone traits. Surprisingly, there was a negative association of free and bioavailable 25(OH)D and some of the bone traits in obese women.

## Introduction

Obesity is a global problem that occurs concomitantly with many other diseases such as insulin resistance and metabolic syndrome. Vitamin D deficiency is prevalent worldwide and has been suggested to be associated with many illnesses, including cancer, autoimmune diseases, hypertension, metabolic syndrome and diabetes [1–4].

Studies have shown that obese individuals have lower serum 25-hydroxyvitamin D concentrations (25(OH)D) than normal weight individuals [5–7]. Wortsman *et al*. concluded that the observed association between vitamin D insufficiency and obesity is likely attributable to decreased bioavailability of vitamin D_3_ from the skin and dietary sources because of its deposition in body fat compartments [5]. Also decreased exposure to sunlight because of limited mobility and negative feedback from elevated 1,25-hydroxyvitamin D (1,25(OH)_2_D_3_) and PTH concentrations on hepatic synthesis of 25(OH)D have been proposed as possible reasons for lower 25(OH)D concentrations among obese people [8]. PTH is a key regulator of calcium balance in the body and according to literature the serum PTH concentration may rise when 25(OH)D values fall below 40–60 nmol/L [9]. Increased serum PTH causes an increase of bone turnover [10].

25(OH)D is transported bound to vitamin D binding protein (DBP) in circulation. DBP transports 25(OH)D from the liver to the kidneys and other tissues and binds 85–90% of the total circulating 25(OH)D and 85% of the total circulating 1,25(OH)_2_D_3_. Albumin and lipoproteins bind the remaining 15%, but with a lower affinity. Less than 1% of the vitamin D metabolites are free in circulation [11]. DBP is suggested to also have a role in bone formation. In its deglycosylated form, DBP can act as a macrophage activating factor and produce morphological changes in osteoclasts and also activate them, which is correlated with enhanced bone resorption [12]. DBP has an important role in binding with 25(OH)D and 1,25(OH)D as well as in regulating their concentrations and functions.

According to the free hormone hypothesis only hormones that are not bound to transporting proteins can enter the cells and have actions there [13]. 25(OH)D is commonly known to enter renal cells in a 25(OH)D-DBP complex via a megalin-mediated receptor. However, most other tissues are exposed to free 25(OH)D (25(OH)D_Free_) or bioavailable (free+albumin bound) 25(OH)D (25(OH)D_Bio_). There has been discussion whether these forms could be better markers of vitamin D status [14–16]. Both the concentration of DBP and the affinity of 25(OH)D to DBP influence the concentrations of 25(OH)D_Free_ and 25(OH)D_Bio_.

As 25(OH)D is low in obese subjects, one could expect that this would influence bone health negatively. Yet, studies have shown the reverse: a high BMI correlates positively with areal bone mineral density (BMD) [17]. It is a common notion that obesity is associated with stronger bones and prevents osteoporosis and fractures, hip fractures in particular but not upper extremity fractures [18]. Besides additional loading because of greater body weight on the weight-bearing skeleton, adipose tissue is likely to produce more estrogen which, in turn, has a major impact on female skeleton in particular [19]. Excess weight in adolescence is known to be associated with larger bone cross-section and to modulate also BMD in adulthood [20]. Apparently, physical activity during adolescence and later in life played a role in this respect. It is, however, recalled here that the body weight per se is not the major determinant of loading, but the intensity and amount of physical activity is. When the amount of excess body is compared to activity-induced forces corresponding to multiples of body weight [21], the role of static body weight becomes secondary. Therefore it is important to study associations in skeletal sites that are affected and not affected by habitual loading.

To our knowledge, the direct association between the free forms (25(OH)D_Free_ and 25(OH)D_Bio_) and bone traits in obese subjects has not been studied. The purpose of this study was to explore the association between obese, overweight and normal-weight Finnish women and men in total 25(OH)D, 25(OH)D_Free_, 25(OH)D_Bio_, DBP, and PTH concentrations. In addition, we investigated whether a link exists between serum 25(OH)D, 25(OH)D_Free_, and 25(OH)D_Bio_ concentrations and skeletal status of weight-bearing and non-weight bearing bones as measured with peripheral quantitative computed tomography (pQCT) and biochemical markers of bone metabolism.

## Materials and methods

### Ethics statement

All subjects gave their written informed consent to procedures that were conducted in accord with the Helsinki Declaration. The study protocol was approved by the Helsinki Uusimaa Hospital District Ethics Committees.

### Study population

The population-based study was conducted in January-May 2010 (blood sampling in January or March and pQCT-measurements in January-May) and was performed in the Helsinki area (60°N). The subjects comprised of 37- to 47-year-old Caucasian females and males. Recruitment and the study protocol are described in detail elsewhere [22]. Pregnant women were excluded from the study. The total number of recruited participants for the first phase i.e. blood sampling was 678 and of these, 653 participated in the second phase i.e. pQCT measurements. Morbidly obese subjects (BMI ≥ 40) were not included in the analyses (N = 14). In the 25(OH)D, PTH and DBP analyses 58 participants were not included due to incomplete data, or frequent sunbed use (> 10 times during 2008–2010). In pQCT-analysis, the women who reported that their menstruation had ended permanently, were excluded. Unfortunately we did not have information if they were menopausal. Also subjects with earlier history of eating disorder or medication affecting calcium or bone metabolism were excluded. The total number of subjects included in the pQCT-analysis was 554.

### Dietary intake and background data collection

The dietary intakes of vitamin D and calcium during the preceding month were evaluated using a validated Food Frequency Questionnaire covering over 70 foods [23]. The subjects completed a questionnaire on medical history, medications and overall health, use of vitamin D and calcium supplements, and physical activity (expressed as weekly minutes engaged in supervised and unsupervised exercises). Holidays spent in sunny locations during winter 2009–2010, from November 2009 to January 2010 or from November 2009 to March 2010 (depending on the time of the blood sampling), served as a measure of sunshine exposure. Sunny locations were defined as locations with a possibility for exposure to UV-irradiation. Smoking was evaluated as pack years and it was calculated by multiplying the number of packs of cigarettes smoked per day by the number of years the person has smoked. Weight and height were measured in light clothing without shoes, and BMI was calculated according to the following formula: weight in kilograms divided by the square of the height in meters (kg/m^2^). Subject were classified according to their BMI as normal-weight (18.5–24.9 kg/m^2^), overweight (25–29.9 kg/m^2^), or obese (30–39.5 kg/m^2^) [24].

### Biochemistry

Twelve-hour fasting blood samples were collected on the first visit. All samples were obtained between 7:30 and 9:15 a.m., and serum was separated by centrifugation and stored immediately after sampling at −20°C or −70°C until analysis. Blood samples were analyzed in one batch in each analysis. Serum S-25(OH)D, albumin and PTH concentrations were analyzed at the Department of Food and Environmental Sciences, University of Helsinki, in 2010. Serum albumin was analyzed by a photometric method by Konelab20 automatic analyzer (Thermo Clinical Labsystems, Espoo, Finland). Inter and intra coefficient of variations (CV%) were <4.6% for the above-mentioned analyses. Serum 25(OH)D concentrations were analyzed by using an IDS enzyme immunoassay kit (Immunodiagnostics Systems Ltd., Boldon, UK). Inter and intra CV%s were 2.7% and 3.2%, respectively. At the time that the samples were analyzed, the laboratory was in the process of achieving the Vitamin D External Quality Assessment Scheme certificate, DEQAS (deqas.kpmd.co.uk/), for ensuring reproducibility of analyses. The laboratory received the DEQAS proficiency certificate for this method in 2012. Serum PTH concentrations were analyzed by using an immunoluminescence-based method by Immulite1000 (Siemens Healthcare Diagnostics, NY, USA). Inter CV% was 8.0% and intra CV% <5.5%. Serum intact pro-collagen type I amino-terminal propeptide (PINP) and serum collagen type 1 cross-linked C-terminal telopeptide (CTX) were analyzed by using an IDS-iSYS Multidiscipline Automated Analyzer (Immunodiagnostic Systems Ltd., Bolton, UK) at the NordLab Oulu and at the Department of Clinical Chemistry of the University of Oulu in 2012. For both assays, intra CV% was <5.3% and inter CV% <2.9%. Total serum osteocalcin was analyzed with a two-site immunoassay-method based on monoclonal antibodies at the Department of Cell Biology and Anatomy, Institute of Biomedicine, University of Turku, Turku, Finland in 2012 described in detail previously [25, 26].

### DBP analysis

DBP concentrations were measured from plasma samples using a commercially available enzyme-linked immunosorbent assay (ELISA; Immundiagnostik AG, Bensheim, Germany) at the Department of Food and Environmental Sciences, University of Helsinki in 2013. This method uses polyclonal DBP antibodies. This enzyme immunoassay is a sandwich assay for the quantitative determination of DBP in serum, plasma and urine samples. The wells of the microtiter plate are coated with polyclonal anti-DBP antibodies.

### Calculation of 25(OH)D_Free_ and 25(OH)D_Bio_

Calculation of 25(OH)D_Free_ was performed with a previously published equation [11,16]. T and F are the total and free vitamin D concentrations, respectively, and KALB and KDBP are the affinity constants for 25(OH)D with albumin and DBP. The affinity constant used for albumin was 6 x 10^5^ and for DBP 7 x 10^8^

$$\left[F\right]==\frac{\left(\text{T}\right)}{\left(1+\text{KALB}\left(\text{ALB}\right)+\text{KDBP}\left(\text{DBP}\right)\right)}$$

25(OH)D_Bio_ was calculated as a sum of 25(OH)D_Free_ and albumin-bound 25(OH)D.

### pQCT measurements

Distal and shaft sites of the non-weight-bearing radius and weight-bearing tibia were measured with pQCT (XCT 2000, Stratec Medizintechnik GmbH, Pforzheim, Germany). Bone density measurements were conducted at the Department of Food and Environmental Sciences, University of Helsinki, in 2010.

The distal sites of the non-dominant radius and left tibia were scanned at 4% and 5% from the distal endplate, respectively. The shaft sites of the radius and tibia were scanned at 30% from the distal endplate. The measurement protocol is described in detail in Laaksonen et al. [27]. Because weight and height are strong determinants of bone-size related traits, we chose to include only the bone traits that are independent of body size [28]. For the distal and shaft sites, volumetric trabecular density (TraD) and cortical density (CorD) values were determined. In addition, cortical strength index (CSI), indicating cortical stability, was calculated as the ratio of cortical bone area to total bone area from both distal and shaft sites of the radius and tibia. For the radius, CV% was 1.6% for TraD and 0.5% for CorD. For the tibia, the corresponding CV%s were 0.5% and 0.6%.

### Statistical analyses

Descriptive crude data are reported as means ± SD and adjusted data as means ±SE. Association between variables of interest was tested with Pearson correlation. If outliers were detected, Spearman correlation was used instead. Background characteristics were compared between the three groups with analysis of variance (ANOVA). The difference between the normal-weight, overweight and obese subjects in 25(OH)D, 25(OH)D_Free_, and 25(OH)D_Bio_, PTH, and DBP concentrations were tested with analysis of covariance (ANCOVA) using vitamin D intake, holidays in sunny locations (yes/no) and age (25(OH)D, 25(OH)D_Free_ and 25(OH)D_Bio_ analyses), Ca intake and age (PTH analysis), or use of hormonal contraceptives (DBP analysis, women) or age (CTX, PINP, osteocalcin analysis) as covariates. Post-hoc comparisons were made with Bonferroni correction.

To determine the factors that affect the measured peripheral bone traits, a backward regression analysis was performed. Men and women were analyzed separately. In these regression models, physical activity, age, pack-years, 25(OH)D, 25(OH)D_Free_, or 25(OH)D_Bio_ were included as independent variables. The model with the largest adjusted coefficient of determination (R^2^) is presented. All analyses were performed using SPSS 24. A P-value of less than 0.05 was considered significant.

## Results

### Characteristics of participants

The mean±SD 25(OH)D concentration in all the subjects was 55.1±19.2 nmol/L and vitamin D and Ca intakes 14.9±13.4 μg/day and 1252±530 mg/day, respectively. The background characteristics of the subjects stratified by sex and BMI groups of normal-weight, overweight, and obese are presented in Table 1. The values in the Table 1 were compared with ANOVA. Obese women had 4.9 μg lower vitamin D intake than normal-weight women (P = 0.019), but Ca intake did not differ between the groups (P = 0.603). They also had over an hour less physical activity (P = 0.045) and two years more pack-years than normal-weight women (P = 0.045). In obese men, vitamin D and Ca intakes were 5.7 μg and 324 mg higher (P = 0.018, and 0.003, respectively) and they had smoked in average five years more than their normal-weight peers (P = 0.009). Mean vitamin D intake reached the recommendations of the Nordic European countries (10 μg/day) in all groups [29]. 25(OH)D concentration was below 50 nmol/L in 57% of obese women and 59.5% of men. In normal-weight women 35.5% and 44% men had 25(OH)D concentrations below 50 nmol/L.

**Table 1:** Characteristics of the subjects stratified by sex and BMI.

Women				Men				
	Normal-weight N = 186	Over-weight N = 130	Obese N = 68	P	Normal-weight N = 56	Over-weight N = 72	Obese N = 36	P
**Demographics**								
Age (years)	41.8±2.7	42.1±2.7	42.0±2.8	0.471	42.1±3.1	42.2±3.2	42.5±2.8	0.493
Height (cm)	166±6.5	165±5.5	163±7.1	0.001	180±7.2	180±6.5	178±4.8	0.092
Weight (kg)	62±6.4	73.7±6.5	89±10	<0.001	74.7±7.0	87.4±7.2	102.8±9.7	<0.001
BMI (kg/m^2^)	22±1.6	27±1.5	33±2.7	<0.001	23.0±1.40	27.0±1.36	32.6±2.30	<0.001
**Background**								
Vitamin D intake (μg/day)*	16.2±14	15.1±13	11.1±7	0.019	11.3±6.10	16.4±16.9	18.4±19.2	0.061
Calcium intake (mg/day)*	1238±537	1281±515	1207±489	0.603	1121±461	1291±516	1445±661	0.003
Physical activity (min/week)	264±270	257±203	201±186	0.045	196±178	199±148	229±353	0.717
Holidays in sunny locations**	31%	16%	4%		9%	14%	4%	
Smoking (pack-years)	2.7±6.2	3.9±6.8	4.8±6.7	0.045	3.9±9.5	5.6±9.5	9.3±11.6	0.026
**Blood concentrations**								
DBP (mg/L)	358±89	375±92	398±102	0.007	357±79.4	362±74.2	377±81.6	0.463
25(OH)D (nmol/L)	58.4±19.5	56.9±21.2	50.1±17.6	0.002	54.5±17.3	54.9±19.2	49.7±18.9	0.174
25(OH)D_Free_ (pmol/L)	13.1±4.7	12.4±5.7	10.0±3.9	<0.001	12.1±4.5	11.9±4.8	10.6±4.7	0.271
25(OH)D_Bio_ (nmol/L)	5.2±1.9	4.90±2.3	3.8±1.5	<0.001	4.9±1.88	4.8±1.79	4.3±1.87	0.232
Albumin (g/L)	43.4±2.7	43.0±3.0	42.2±2.7	0.012	45.3±2.86	45.0±3.2	45.0±2.41	0.811
PTH (ng/L)	53.3±24.6	57.3±26.1	62.3±27.7	0.042	50.9±20.9	50.3±24.4	55.3±22.6	0.493
CTX (ng/mL)	0.37±0.16	0.33±0.15	0.29±0.13	0.001	0.54±0.18	0.49±0.19	0.39±0.12	<0.001
PINP (ng/mL)	36.7±14	35.2±13	32.6±12	0.1	43.9±13	40.7±14	37.1±11	0.036
Osteocalcin (ng/mL)	7.8±2.7	7.4±2.7	6.3±2.3	0.001	9.14±2.4	8.3±2.6	7.3±2.0	0.001
**pQCT traits**	N = 168	N = 120	N = 62		N = 65	N = 90	N = 42	
Distal radius TraD (mg/cm^3^)	192±29.0	198±26.5	206±28	0.004	219±28	232±26	228±26	0.012
Distal radius CSI	0.25±0.06	0.25±0.05	0.26±0.06	0.555	0.28±0.06	0.27±0.05	0.27±0.06	0.853
Radial shaft CorD (mg/cm^3^)	1148±33	1133±39	1128±41	<0.001	1124±49	1117±39	1109±34	0.187
Radial shaft CSI	0.86±0.07	0.87±0.04	0.87±0.55	0.464	0.85±0.05	0.85±0.05	0.86±0.04	0.704
Distal tibia TraD (mg/cm^3^)	209±30	219±24	223±25	<0.001	225±27	243±28	237±28	<0.001
Distal tibia CSI	0.21+0.06	0.22±0.04	0.24±0.05	0.022	0.24±0.05	0.27±0.07	0.27±0.06	0.029
Tibial shaft CorD (mg/cm)^3^	1110±29	1102±26	1100±28	0.008	1106±25	1088±26	1094±28	<0.001
Tibial shaft CSI	0.80±0.05	0.80±0.05	0.81±0.05	0.230	0.80±0.05	0.82±0.04	0.81±0.04	0.019

*Food and supplements.

**% of participants spent holidays in sunny locations. All values are means; ± SD, ANOVA. Abbreviations: 25(OH)D_Free_, Free 25(OH)D; 25(OH)D_Bio_, Bioavailable 25(OH)D; CTX, serum collagen type 1 cross-linked C-terminal telopeptide; PINP, serum intact pro-collagen type I aminoterminal propeptide; TraD, trabecular density; CSI, Cortical strength index; CoD. cortical density.

### Differences in 25(OH)D, 25(OH)D_Free_, and 25(OH)D_Bio_ concentrations between BMI groups

25(OH)D and 25(OH)D_Free_ correlated negatively with PTH in both men (r = -0.179, r = -0.159, P = 0.013, and 0.042, respectively) and women (r = -0.232; P<0.001, and r = -0.211; P<0.001, respectively). 25(OH)D_Bio_ correlated negatively with PTH in women (r = -0.207; P<0.001), but in men the correlation did not reach significance. Total, free, and bioavailable 25(OH)D correlated inversely with BMI in women and when both sexes were analyzed together (r = -0,20; -0,21; -0,23, respectively, P<0.001) and (r = -0.15; -0.19; -0.19; respectively, P<0.001), but not in men.

**Fig 1:**
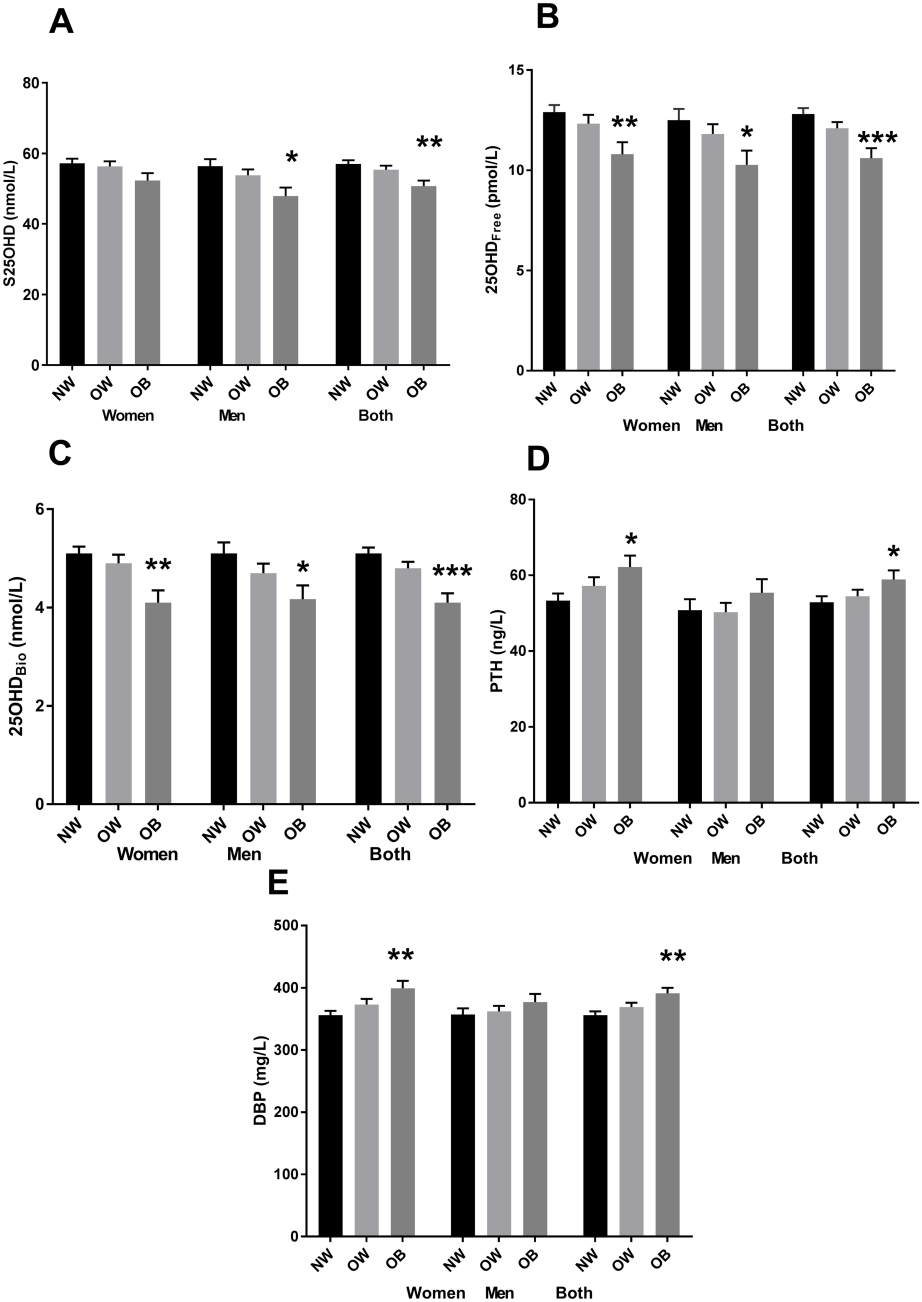
Differences in total (A), free (B) and bioavailable 25(OH)D (C), PTH (D) and DBP (E) concentrations between normal weight, overweight and obese women, men and both sexes combined. Results are shown for mean (±SE). The values were adjusted for vitamin D intake, age, and holidays in sunny locations (A,B,C), calcium intake (D) or hormonal contraceptives (E). ANCOVA, Bonferroni pairwise comparisons between normal-weight and obese: *P<0.05,**P<0.01,***<0.001, respectively.

In women 25(OH)D concentrations did not differ among the BMI groups (Fig 1A). In men, 25(OH)D was lower in obese men than in normal-weight (48.0 ±2.4 nmol/L vs. 56.4±2.0 nmol/L, P = 0.003, ANCOVA) but there were no difference between normal weight and overweight group or overweight and obese group Similarly, when both sexes were analyzed together, obese subjects had lower 25(OH)D than normal-weight subjects (50.7±1.6 vs. 57.0±1.0 nmol/L, respectively; P = 0.003, ANCOVA). There was also a significant difference between overweight and obese group (P = 0.023, ANCOVA). 25(OH)D_Free_ and 25(OH)D_Bio_ concentrations were lower in the obese women (10.8±0.6 vs. 12.9±0.4 pmol/L and 4.1±0.3 nmol/L vs. 5.1 ±0.1 nmol/L, P<0.008 and <0.003, respectively, ANCOVA)(Fig 1B and 1C). Also in men, 25(OH)D_Free_ and 25(OH)D_Bio_ were lower in obese subjects (10.3±0.7 vs. 12.5±0.6 pmol/L and 4.2±0.3 vs. 5.1±0.2 pmol/L, P = 0.044 and 0.032, respectively, ANCOVA). When both sexes were analyzed together, significant difference remained (P<0.001, ANCOVA). There was also a significant difference between overweight and obese in 25(OH)D_Free_ and 25(OH)D_Bio_ (ANCOVA). There were no difference between normal weight and overweight group or overweight and obese group in women and men analyzed separately (ANCOVA).

### Differences in PTH and DBP concentrations between BMI groups

DBP correlated positively with BMI when men and women were analyzed together (r = 0.363, P = 0.025). In women, DBP and PTH correlated positively with BMI (r = 0.129, p = 0.016; r = 0.181, p = 0.000, respectively). In women, PTH was higher in obese compared to normal-weight subjects (62.2±3 ng/L vs. 53.3±1.9 ng/L, P = 0.045, ANCOVA) (Fig 1D). In men, the difference was not significant. When both sexes were analyzed together, the difference was significant (P = 0.047, ANCOVA). Obese women had higher DBP concentration than normal-weight women (399±12mg/L vs. 356±7 mg/L, P = 0.008, ANCOVA) (Fig 1E). In males, DBP concentration did not differ among BMI groups. When both sexes were analyzed together, DBP was higher in obese subjects compared to normal-weight subjects (391±9 vs. 356±6 mg/L, P = 0.004, ANCOVA). There was no difference in PTH or DBP concentrations between normal-weight and overweight and overweight and obese subjects.

### Differences in bone traits and bone turnover markers between BMI groups

Mean ±SD bone traits and comparison between normal-weight, overweight and obese subjects are shown in Table 1. Trabecular density in obese women was 6.3% higher in distal radius and 6.6% higher in distal tibia compared to normal-weight women (P = 0.004 and P<0.001, respectively). In addition, CSI was 12.5% higher in obese women compared to normal weight women (P = 0.022). Cortical density was 1.7% and 0.9% lower in obese women in the shaft sites of radius and tibia (P<0.001; P = 0.008, respectively). (ANOVA, Table 1).

In obese men, trabecular density was 3.9% higher in distal radius and 12% higher in distal tibia than in normal-weight men (P = 0.012 and P<0.001). (ANOVA, Table 1). Distal tibia CSI was 11% higher and tibial shaft CSI was 1% higher (P = 0.029; P = 0.019, respectively). Tibial shaft cortical density was 1% lower in obese men compared to normal weight men (P<0.001).

The mean±SD crude values of bone turnover markers (CTX, PINP and osteocalcin) are shown in Table 1. After adjusting with age, CTX and PINP were lower in obese women compared to normal weight (mean±SE) (0.38±0.17 vs. 0.29 ±0.13, P< 0.001, ANCOVA). Also PINP and osteocalcin were lower in the obese women (37.1±15 vs. 32.7±11; 7.9±2.8 vs. 6.2 ± 2.3, P = 0.034 and <0.001, respectively, ANCOVA. In men, CTX and osteocalcin were lower in obese compared to normal weight men (0.54 ± 0.17 vs. 0.39±0.12, P<0.001; 9.1±0.24 vs. 7.4±2.0, P = 0.001, respectively, ANCOVA).

### Associations of total, free and bioavailable 25(OH)D measures with bone traits in BMI groups

To determine the factors associated with pQCT-measured bone traits in normal-weight and obese women and men, multiple linear regression analyses were performed. Physical activity, smoking and age were included as independent variables each analysis. Depending on the model, 25(OH)D, 25(OH)_Free_ or 25(OH)D_Bio_ were included as independent variables. The results are shown in Table 2. In obese women, 25(OH)D was a negative determinant of distal radius TraD (R^2^ = 0.089, β = -0.346, P = 0.009) and tibial shaft CSI (R^2^ = 0.146, β = 0.372, P = 0.004). Furthermore, 25(OH)D_Free_ was a negative determinant of distal radius CSI (R^2^ = 0.070, β = -0.255, P = 0.049), and tibial shaft CSI (R^2^ = 0.113, β = -0.317, P = 0.012). In addition, 25(OH)D was a negative determinant of radial shaft CoD (R^2^ = 0.050, β = -0.255, P = 0.045). 25(OH)D_Bio_ was a negative determinant of distal radius CSI (R^2^ = 0.072, β = -0.260, P = 0.045), and tibial shaft CSI (R^2^ = 0.107, β = -0.308, P = 0.015). 25(OH)D_Bio_ was a negative determinant of radial shaft cortical density (R^2^ = 0.059, β = -0.273, P = 0.032). In normal-weight and overweight women, no associations were found between bone traits and 25(OH)D, 25(OH)D_Free_ or 25(OH)D_Bio_ concentrations. Both in normal-weight and obese men, no associations were found between the 25(OH)D concentrations and bone traits. In overweight men, there was a positive association between total 25(OH)D and distal radius CSI (p = 0.046, β = 0.208).

**Table 2:** Standardized β-coefficients and R^2^ -values in backward linear regression model for determinants of pQCT-bone traits in obese women.

	25(OH)D			25(OH)D_Free_			25(OH)D_Bio_		
Obese women N = 62	Adjusted R^2^	β	P	Adjusted R^2^	β	P	Adjusted R^2^	β	P
**Distal radius TraD (mg/cm**^**3**^**)**	0.089^a^	-0.346	0.009	0.027	-0.214	0.100	0.016	-0.189	0.147
**Distal radius CSI**	0.028	-0.152	0.243	0.070^c^	-0.255	0.049	0.072^f^	-0.260	0.045
**Radial shaft CoD (mg/cm^3^)**	0.016	-0.180	0.162	0.050^d^	-0.255	0.045	0.059^g^	-0.273	0.032
**Tibial shaft CSI**	0.146^b^	-0.372	0.004	0.113^e^	-0.317	0.012	0.107^h^	-0.308	0.015

Other determinants in the models were age, physical activity, and smoking.

TraD trabecular density, CSI Cortical strength index, CoD cortical density, 25(OH)D_Free_ = Free 25(OH)D, 25(OH)D_Bio_ = Bioavailable 25(OH)D.

Other remaining variables;

^a^physical activity (P = 0.124),

^b^age (P = 0.058),

^c^smoking (P = 0.262) and age P = 0.141,

^d^ -, ^e^ age (P = 0.074) and physical activity (P = 0.172),

^f^age (P = 0.146) and smoking (P = 0.281),

^g^P = 0.032,

^h^age (P = 0.079) physical activity (P = 0.169).

## Discussion

In this cross-sectional study of 37- to 47-year-old men and women, we found that in the total population and in men, 25(OH)D, 25(OH)D_Free_, and 25(OH)D_Bio_ were lower in obese than in normal-weight persons. The difference in 25(OH)D did not reach statistical significance in women. In addition, DBP and PTH were higher in obese than normal-weight women and in the total population. In men, no difference in DBP and PTH was found between the BMI groups. We also found that in obese women, there was a weak negative association between 25(OH)D, 25(OH)_Free_ and 25(OH)_Bio_ and some bone traits. Bone turnover markers were lower in obese subjects.

It is well established that serum 25(OH)D is lower in obese people and inversely correlated with BMI [6, 8, 30]. Evidence suggest that as lipophilic substance, vitamin D is trapped or sequestered in adipocytes and can only be released when there is net mobilization of fatty acids in the triacyl glycerol droplet. Therefore it has been speculated that in obese individuals with larger volume of fat tissue less vitamin D could be available for liver synthesis into 25(OH)D [5, 6, 31]. Also the volume of other tissues, i.e. blood and muscles, where 25(OH)D is also distributed, is larger in obese persons. In addition, some studies have shown that obesity attenuates the rise of 25(OH)D in the circulation after UV exposure and that the 25(OH)D response to oral vitamin D dosing is BMI-dependent [32, 33]. In our study, we were able to find significant differences in 25(OH)D between BMI groups only in men and in total population, although a similar trend was seen also in women. Despite adequate vitamin D intake in all of the BMI groups, over 50% of obese women and men had 25(OH)D lower than 50 nmol/L.

According to the free hormone hypothesis, the unbound form of 25(OH)D would correlate better with the biological actions of vitamin D than the bound form [13]. Should this hypothesis be true, circulating unbound 25(OH)D would enter the extrarenal tissues passively, and local 1α-hydroxylase would convert it to the active form of 1,25OH_2_D_3_. Studies on 25(OH)D_Free_ in obese individuals are few and the results have been controversial. The method for determining the concentrations of 25(OH)D_Free_ and 25(OH)D_Bio_ in these studies has varied from estimated values [34] to direct measurement [17, 35]. The calculation of 25(OH)D_Free_ concentrations is based on mathematical formula which takes into account total 25(OH)D, DBP and albumin concentrations as well as the corresponding affinity constants. 25(OH)D_Free_ can also be measured directly by equilibrium dialysis, ultrafitration or immunoassays. A novel direct twostep immunoassay (liquid chromatography-tandem mass spectrometry, LC-MS/MS) for measuring 25(OH)D_Free_ has shown good correlations with calculated or directly measured values, at least in normal weight subjects [14]. According to Malmström et al. when the 25(OH)_Free_ concentration is estimated by calculation, the assays to measure DBP and total 25(OH)D must be the best methods available, such as LC-MS/MS (for total 25(OH)D levels) and polyclonal antibody–based immunoassays (for measuring DBP) [36]. The limitation in our study is that we were not able to directly measure 25(OH)D_Free_. However, the total 25(OH)D concentration, used in the calculation of 25(OH)D_Free_ and 25(OH)D_Bio_, was measured with enzyme immunoassay in a laboratory that was in the process of achieving DEQAS-certificate and DBP was measured with polyclonal antibody-based assay and therefore the calculation of 25(OH)D_Free_ and 25(OH)D_Bio_ can be considered reliable. According to Bikle et al. experience with direct measurements of the free levels is limited and conclusions cannot be made on the influence of racial differences and the impact of inflammatory or other disease states that may alter the relationship between total and free metabolite levels [37].

A study of 22- to 45-year-old obese and normal-weight women in Sweden discovered that obese women had lower calculated 25(OH)D_Free_ than normal-weight women [34]. The authors also reported lower 25(OH)D concentrations among obese women. Similarly, in a study conducted in the United Kingdom, measured 25(OH)D_Free_ and 25(OH)D_Bio_ were lower in obese men and women than in normal-weight or overweight subjects aged 25–75 years [17]. Also 25(OH)D_3_ was lower in obese and overweight people than in normal-weight people in the fall and spring, but not in the winter, and correlated negatively with whole-body fat mass in all seasons. The reason for not seeing differences in the winter may be due to the fact that obese people have similar cutaneous synthesis of vitamin D, but the rise of 25(OH)D in serum is blunted [38, 39]. Our results on 25(OH)_Free_ are consistent with previous studies. Obese subjects appear to have lower 25(OH)_Free_ concentrations regardless of the method used for the measurement. 25(OH)D was lower among obese subjects only in men and when men and women were analyzed together. One reason for not having such a clear differences in 25(OH)D could be the same as in the study made in United Kingdom since our study was conducted in winter/early spring, when UVB-induced endogenous vitamin D synthesis in the skin is marginal in Finland. The lack of vitamin D levels collected during summer months may have narrowed the range of 25(OH)D data and reduced the strength of observed associations.

PTH has been suggested to be a health outcome reference for optimal vitamin D status. If vitamin D intake is low, and gut calcium absorption is therefore reduced and 25(OH)D concentration is low enough, PTH is expected to rise [40]. As expected, we found an inverse association between 25(OH)D and PTH. Furthermore, in our study, PTH differed between the BMI groups, but only in women and when the sexes were combined. Contrary to our results, Walsh *et al*. [17] reported that PTH did not differ by BMI group and did not correlate with BMI in either sex. They speculated that the relation between 25(OH)D and PTH may be altered in obesity. In some studies, body weight was a strong predictor of PTH, but 25(OH)D had little or no relation with PTH. Obese young Finnish adults exhibited lower total and measured 25(OH)D_Free_ and slightly higher PTH than their normal-weight peers, and 25(OH)D_Free_ was associated with obesity-related parameters [35].

Earlier studies have had conflicting results on the relationship between BMI and DBP. Some studies have shown no association between DBP and BMI [17, 35, 41]. Taes et al. [42] found a positive correlation between DBP concentrations and BMI and leptin concentrations and speculated that the relation DBP-fat mass could also have influenced glucose handling. In Karlsson et al. study, DBP was higher in obese subjects [34]. Since estrogens are known to increase DBP concentrations, they speculated that although estrogen levels are not normally elevated in obesity, higher levels of free estrogen in obesity could possibly have an effect on hepatic DBP production. They also suggested inteleukin-6 could be behind the higher DBP concentration in the obese subjects, since in vitro IL-6 has been shown to increase hepatic DBP production [43] and IL-6 is raised in obesity [44].

Also the DBP genotype can influence the measured DBP concentrations as well as on DBP’s ability to bind 25(OH)D. In our previous study of the same study population, we found genetic differences in DBP, 25(OH)D_Free_, and 25(OH)D_Bio_ concentrations [45]. In addition, Almesri *et al*. [46] found an association between DBP gene polymorphism and obesity. In the present study, some of the diplotype groups were too small to make statistical comparisons and therefore we were not able to take into account DBP polymorphism. Also the use of different methods (monoclonal vs. polyclonal) for measuring DBP can affect the results. The optimal method for measuring DBP concentrations has also been under debate. The two different methods are one that use monoclonal antibodies and the other that is using polyclonal antibodies. Method using monoclonal antibodies may not be reliable to measure polymorphic DBP in groups of different races and genotypes because monoclonal assay may quantify DBP concentration differentially by DBP isoform. The polyclonal method is not subject to bias and in contrast to monoclonal method, the polyclonal does not differ by race [47, 48].

Because weight and height are strong determinants of bone-size related traits [28], we evaluated only the bone traits that are independent of body size. We found that 25(OH)D was a significant but weak negative determinant of trabecular density of the distal radius, and 25(OH)D, 25(OH)D_free_ and 25(OH)D_Bio_ were negative determinants of tibial shaft CSI. 25(OH)D_Free_ and 25(OH)_Bio_ were also negatively associated with cortical density of the radial shaft. In normal-weight women, no significant association was found between 25(OH)D, 25(OH)D_Free_, or 25(OH)D_Bio_ and bone traits. Also in men, no associations were found between 25(OH)D concentrations and bone traits. Earlier studies using DXA have shown that calculated 25(OH)D_Free_ and 25(OH)D_Bio_ but not 25(OH)D, correlated with whole-body and hip areal BMD, where the adjustment for 25(OH)D_Free_ and 25(OH)D_Bio_ values with DBP genotype-specific coefficients improved the association [15]. In our study, obese men and women had higher trabecular density and CSI but slightly lower cortical density. Furthermore obesity was associated with lower concentrations of bone turnover markers in both men and women. Walsh *et al*. discovered higher whole-body, lumbar spine, and hip areal BMD with DXA, and distal radius and tibia trabecular density measured with HR-pQCT in obese and overweight groups than in the normal-weight group. Also bone turnover was higher in the obese group, contrary to our results. However, Walsh did not look at the direct association of total, free and bioavailable 25(OH)D with bone parameters [17]. They suggested that in their study, the lower serum 25(OH)D in obesity reflected true vitamin D deficiency, but that possibly the adverse skeletal effects are countered by positive skeletal effects of obesity such as increased estrogen synthesis from adipocyte aromatase or adipocyte hormones such as leptin. However, in our study women were premenopausal and therefore the estrogen production by the ovaries exceeds that from adipose tissue. A potential explanation for the negative associations of free and bioavailable 25(OH)D concentrations with some bone traits in obese women of our study, could be the altered vitamin D metabolism due to higher DBP and lower 25(OH)D_Free_ and 25(OH)D_Bio_ in obese women. Obese women also had more smoking years and they exercised less than the normal weight women.

Strengths of our study are the large population-based sample of both women and men, assessment of several bone traits with pQCT in two functionally different bones (non-weight bearing radius and weight bearing tibia) and sites (mostly trabecular distal site and cortical diaphysis), analysis of several established biomarkers, and extensive background data. Moreover, in contrast to commonly used dual-energy x-ray absorptiometry, providing aBMD values that are difficult to interpret unambiguously [49]. pQCT provides relevant data for trabecular and cortical BMD as well as bone geometry, size, and mass [50]. However, measurement of fat percentage, waist circumference or body composition would have been a better proxy for obesity than BMI. We neither did not specifically evaluate the confounding influence of bone-loading activity on bone traits or take the history of physical activity into account.

## Conclusion

The observed associations between BMI and 25(OH)D, 25(OH)D_Free_, and 25(OH)D_Bio_, DBP, and PTH suggest that obese subjects may differ from normal-weight subjects in their vitamin D metabolism. BMI associated positively with trabecular bone traits and CSI in our study, and slightly negatively with cortical bone traits. Surprisingly, there was a negative association of free and bioavailable 25(OH)D and some of the bone traits in obese women.

## Supporting information

S1 Table (XLSX)
